# Medical Transport for 769 COVID-19 Patients on a Cruise Ship by Japan Disaster Medical Assistance Team

**DOI:** 10.1017/dmp.2020.187

**Published:** 2020-06-05

**Authors:** Hideaki Anan, Hisayoshi Kondo, Ichiro Takeuchi, Tomoki Nakamori, Yu Ikeda, Osamu Akasaka, Yuichi Koido

**Affiliations:** Emergency Medical Center, Fujisawa City Hospital, Japan; Japan DMAT Secretariat, National Hospital Organization Disaster Medical Center, Tokyo, Japan; Advanced Critical Care and Emergency Center, Yokohama City University, Japan; Emergency Medical Center, Yokohama Rosai Hospital, Japan

**Keywords:** disaster medicine, infectious disease medicine, quarantine

## Abstract

The *Diamond Princess* cruise ship, carrying 3711 passengers and crew members, docked at Yokohama Port in Japan on February 3, 2020. A quarantine was immediately instituted because 1 passenger who had disembarked in Hong Kong was confirmed to have tested positive for coronavirus disease 2019 (COVID-19). After the quarantine began, all passengers and crew were tested using the severe acute respiratory syndrome coronavirus-2 (SARS-CoV-2) polymerase chain reaction assay on the ship, 696 of whom tested positive. In total, 769 patients, including 696 with COVID-19, required transport to a hospital. The Japan Disaster Medical Assistance Team (DMAT) successfully picked up and safely transported the COVID-19 patients using a novel classification system to prioritize patients. The Japan DMAT transported 203 patients to hospitals in Kanagawa and another 566 patients to hospitals in 15 different prefectures.

This study describes the response to an outbreak of coronavirus disease 2019 (COVID-19) on a cruise ship off the coast of Japan. On January 20, 2020, the *Diamond Princess* cruise ship departed on a round trip voyage from Yokohama, Japan, carrying approximately 3700 passengers and crew members. It returned to Yokohama Port by means of domestic and international ports on February 3. A quarantine was instituted immediately that day because 1 passenger who had disembarked in Hong Kong on January 25 was confirmed to have tested positive for COVID-19. Two days later, pharyngeal swabs of 31 passengers were tested for severe acute respiratory syndrome coronavirus-2 (SARS-CoV-2) by polymerase chain reaction (PCR) assay, 10 of which came back positive. As a result, the Japanese government decided to quarantine the passengers and crew members on the ship for 14 days. All passengers were confined to their cabins as of February 5.

## MANAGEMENT

### PCR Assay of SARS-COV-2 to Determine the Need for Quarantine

The Japanese government decided to check all passengers and crew members with symptoms of COVID-19, such as fever, cough, and sore throat, as well as the passengers who had close contact with the individuals who had tested positive for COVID-19. SARS-CoV-2 PCR assays were performed from February 4 to 25. The test was performed on 6 to 831 samples per day until all 3711 passengers and crew were tested. The number of people confirmed to have COVID-19 was between 3 and 99 people per day, reaching 696 people in total.

### Cooperation With the Japanese Government

Japanese law prescribes that the city and prefecture where the port is located are responsible for situational management. Because the port where the ship was docked was located in Yokohama, in Kanagawa Prefecture, local governments were in charge of managing the situation. However, similar to a disaster, identifying hospitals that can admit a large number of patients and managing medical transport in such situations represents a difficult challenge. Therefore, the Japanese Disaster Medical Assistance Team (DMAT) was contacted for assistance on February 5.

### Medical Transport Management by the Japan DMAT

The Japan DMAT established headquarters in the prefectural government office of Kanagawa and a local coordination office on the ship, and from February 6 to 26, performed medical examinations of the passengers, selected host hospitals, and managed medical transport. A total of 472 DMAT members (157 doctors, 123 nurses, 192 others) participated: 283 engaged in onboard medical care and triage and 189 worked off-board. The Japan DMAT performed daily medical examinations of individuals based on their symptoms and PCR results onboard the ship, and categorized them according to the degree of urgency using the following classifications. Most time-critical patients who required urgent emergency treatment regardless of PCR result were classified into category I-1 and transported immediately to Critical Care and Emergency Medical Centers in Yokohama City. Urgent patients who needed treatment because of a severe underlying disease regardless of PCR result were classified into category I-2 and transported to designated medical institutions (DMIs) specializing in infectious disease in other cities in Kanagawa Prefecture.

Passengers who were at risk of becoming severe if infected with SARS-CoV-2 were classified into category II. To prevent infection, they were then transported to an isolation facility designated by the government after being confirmed to be asymptomatic and to have a negative PCR result. The remaining patients who had a positive PCR result but were asymptomatic or had very mild symptoms, such as sore throat and mild cough, were classified into category III and transported to DMIs specializing in infectious disease in other prefectures.

The Japan DMAT, emergency medical systems, and Japan Self-Defense Forces (JSDF) were in charge of transport. No isolation chamber was used and each staff member wore personal protective equipment (PPE) that were adapted for use against infectious disease and prepared by the government. Patients who required long-distance transport were assigned to the Japan DMAT because only DMAT includes doctors and nurses as members. The longest distance a patient was transported to a hospital was 500 km away from the Port of Yokohama.

## RESPONSE TO THE PROBLEM

Two problems related to patient transport were evident. First, if multiple family members who were passengers on the ship tested positive for SARS-CoV-2, they could not be admitted to the same hospital because the number of isolation beds in each hospital was limited. Second, if some family members were PCR-positive, but others were PCR-negative, it was not easy for the PCR-negative passengers to disembark because of infection control and quarantine law. This also made it difficult for the patients to understand their family member’s situation and caused stress among the attending doctors. Therefore, the Japan DMAT decided to transport patients and their families with or without infection to the same hospital by using general hospital beds and changing legal practices.

### Transportation

**FIGURE 1:**
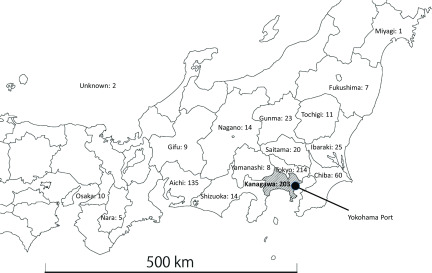
Number of Hospitalized Patients in Each Prefecture.

In accordance with the Japanese Infectious Disease Law, patients with COVID-19 were required to enter a negative pressure chamber. However, only 74 negative pressure chambers were available among 8 hospitals in Kanagawa Prefecture, which was an inadequate number. Therefore, the Japanese government eased the criteria, making it possible for patients with COVID-19 to be admitted to private rooms in general hospitals. After easing these criteria, the number of beds available for patients with COVID-19 in Kanagawa Prefecture increased to around 200; however, this was still inadequate. Therefore, the Japan DMAT and Ministry of Health, Labour, and Welfare asked DMIs in other prefectures to accept the patients with COVID-19. A total of 769 people (731 patients, 38 family members) were transported to hospitals. Among the patients, 108 were classified into category I, 83 into category II, and 540 into category III. Finally, 203 patients were transferred to 37 hospitals in Kanagawa Prefecture, and 566 patients were transferred to 113 hospitals in other prefectures (Figure 1).

In Kanagawa Prefecture, where Yokohama Port is located, 203 patients were transported within the prefecture, and 566 to 15 other prefectures

## DISCUSSION

Quarantines are often implemented at ports as border measures against infectious diseases. The isolation of ship passengers and crew members during a 30- to 40-day incubation period was effective to protect against a plague pandemic in 1337.^1^ Therefore, confinement to a ship such as the *Diamond Princess* has become a standard approach in terms of quarantine. However, ships are increasing in size and carrying more passengers and crew members. Therefore, some changes in terms of quarantine were required. For example, substantial resources were needed to conduct testing on all 3711 people.

A previous study on the management of a ship with an outbreak of influenza virus suggested that outbreaks of infectious disease spread easily on a ship.^2^ However, it was impossible to prepare private isolation rooms for all 3711 individuals on mainland Japan; thus, the passengers and crew members had to be quarantined on the ship. This strategy was considered effective to protect mainland Japan from the spread of SARS-CoV-2.^3^ Therefore, appropriate screening and patient transport are important to control the spread of infectious diseases. Allowing a ship with a large number of infected patients to dock can be an unmanageable burden for local regions where a port is located. A sudden increase in medical needs can be described as a “disaster.” Selecting host hospitals, conducting medical checks on passengers, and managing transport are urgent issues. The Japan DMAT was optimal for managing this situation, as it often manages such tasks after a natural disaster.^4,5^

These disaster experiences were useful for managing the issue concerning the *Diamond Princess*. For example, the Japan DMAT categorized the patients to determine priorities smoothly. On the other hand, the method of patient transport was different than usual. Usually, aircraft transportation is selected for such long distances. As a countermeasure against a future expected Nankai Trough earthquake, which would cause extensive damage, a wide-area medical transportation plan using aircraft from the JSDF has been prepared.^6,7^ In addition, doctor helicopters that carry doctors and nurses are available in Japan.^8,9^ However, helicopters were not permitted to transport infected patients in this case because adequate protection could not be secured. Therefore, all patients with COVID-19 were transferred by land.

Critical care and emergency medical centers in Yokohama accepted the patients with or without COVID-19 who had been aboard the ship and required immediate emergency treatment. However, providing medical care with proper protection is challenging. Managing infectious patients consumes more time and resources. Therefore, it was impossible to treat patients without COVID-19 as usual.^10^

Recently, cruise ships have become increasingly bigger and started carrying more passengers. The docking of such large ships when carrying some passengers with an infectious disease has the risk of overwhelming regional medical care systems. Therefore, measures to manage such situations properly should be prepared. Regarding this case, medical and transport management skills were needed to assess the urgency of patients and identify proper host hospitals.

## CONCLUSIONS

DMAT initiated operations on the ship, devised and implemented a triage system for transport, and facilitated transport throughout a local and regional network of hospitals, while dealing with challenges in logistics, hospital capacity, and isolation protocols.
